# New natural agonists of the transient receptor potential Ankyrin 1 (TRPA1) channel

**DOI:** 10.1038/s41598-020-68013-2

**Published:** 2020-07-08

**Authors:** Coline Legrand, Jenny Meylan Merlini, Carole de Senarclens-Bezençon, Stéphanie Michlig

**Affiliations:** Perception Physiology, Nestlé Research, Route du Jorat 57, CH-1000 Lausanne 26, Switzerland

**Keywords:** Chemical biology, Molecular biology

## Abstract

The transient receptor potential (TRP) channels family are cationic channels involved in various physiological processes as pain, inflammation, metabolism, swallowing function, gut motility, thermoregulation or adipogenesis. In the oral cavity, TRP channels are involved in chemesthesis, the sensory chemical transduction of spicy ingredients. Among them, TRPA1 is activated by natural molecules producing pungent, tingling or irritating sensations during their consumption. TRPA1 can be activated by different chemicals found in plants or spices such as the electrophiles isothiocyanates, thiosulfinates or unsaturated aldehydes. TRPA1 has been as well associated to various physiological mechanisms like gut motility, inflammation or pain. Cinnamaldehyde, its well known potent agonist from cinnamon, is reported to impact metabolism and exert anti-obesity and anti-hyperglycemic effects. Recently, a structurally similar molecule to cinnamaldehyde, cuminaldehyde was shown to possess anti-obesity and anti-hyperglycemic effect as well. We hypothesized that both cinnamaldehyde and cuminaldehyde might exert this metabolic effects through TRPA1 activation and evaluated the impact of cuminaldehyde on TRPA1. The results presented here show that cuminaldehyde activates TRPA1 as well. Additionally, a new natural agonist of TRPA1, tiglic aldehyde, was identified and p-anisaldehyde confirmed.

## Introduction

Transient receptor potential channels (TRP) are a family of nonselective cationic channel expressed at the plasma membrane consisting of 28 mammalian members divided in to 6 sub-families: canonical (TRPC), vanilloid (TRPV), ankyrin (TRPA), melastatin (TRPM), polycystin (TRPP) and mucolipin (TRPML) type^1,2^. A subset of TRP channels, essentially from M and V subfamilies as well as TRPA1, can be classified as thermo-sensitive channels covering together a broad range of sensitivity to temperature going from noxiously cold to warm temperature^3,4^. Upon activation of TRP channels, Ca^2+^ enters into the cells and generate cell depolarization leading to transduction of sensory signal. Even though this subset of TRP members are characterized by their sensitivity to specific ranges of temperature, they can be activated by different other type of stimuli as mechanical forces, generated for example by osmolarity or pressure, and endogenous or exogenous chemicals^1^. Among chemicals, TRP channels are particularly sensitive to potentially irritant ingredients contained in plants and herbs and are responsible for the detection of potential noxious stimuli from the environment. The subset of thermo-sensitive TRP channels is, among other tissues, expressed by sensory neurons of the trigeminal nerve in the oral cavity and participate to the coding of chemesthesis that refers to the multi-modal sensations related to irritation, pain, warmth, cooling, tingling or numbing produced by chemicals essentially from spices and herbs origins^5^. The question has been raised why humans developed an attractive consumption for spicy food producing close to pungent sensations in mouth. One explanation proposed that from an evolutionary point of view, humans might have learnt to like spices because health benefits are associated to their consumption^6^. Confirming the potential health benefits form spices consumption, recent studies associated health beneficial physiological processes to the activation of TRP channel or to specific compounds known to activate TRP channels. Besides being key transducer of sensory features of spicy ingredients in the oral cavity, TRP channels are involved in various other physiological processes in different systems^7^ as metabolism^8,9^, swallowing function^10^, gut motility , thermoregulation^11^ or adipogenesis^8^**.** A major therapeutic strategy is the inhibition of TRP activated pathways, specially TRPV1 and A1, to block inflammation pathway^7,12^ and pain transduction^7^.

In order to exploit the healthy potential of TRP channel stimulation through the ingestion of agonists contained in foodborne plants, one TRP member of particular interest is TRPA1. Health benefits that might be associated to the stimulation or inhibition of TRPA1 are various, as it has been shown to be expressed in numerous tissues as pancreatic β-cells^13^, intestinal enteroendocrine cells^14^, dorsal root ganglia (DRG) sensory neurons^15^ or skin^16^. Moreover, TRPA1 is activated by a broad variety of natural molecules and has been associated to various physiological mechanisms as well as to the pungent, tingling, irritation and burning experience from their consumption^5^.

Different classes of chemical compounds from plants or spices are able to induce TRPA1 activation. TRPA1 can be activated by the covalent binding of electrophile isothiocyanates found for example in wasabi, mustard or horseradish, or the covalent binding of thiosulfinates as diallyl sulfide or diallyl disulfide found in plants such as garlic or onion form the Allium genus. Some unsaturated aldehydes have shown to elicit as well big response of TRPA1^17^. Other aldehydes, irritant compounds of cigarette smoke, activates TRPA1^18^. Other foodborne activators of TRPA1 are found in the family of alkylamides as the non-specific agonist of TRPV1, hydroxa-α-sanshool, from Szechuan pepper^17^. Finally, members of the vanilloids have also been reported to activate TRPA1. Indeed, even though vanilloids have been initially reported to be canonical agonists of the TRPV sub-family, compounds like ethyl-vanillin^19^ or 6-shogaol or 6-paradol^20^ activate TRPA1. One particular TRPA1 agonist, cinnamaldehyde, is reported to impact metabolism. Cinnamaldehyde, the principal constituent of cinnamon oil, has shown positive impact on insulin sensitivity^21^ and liver fat of obese mice^22^ as well as lowering blood glucose level in diabetic mice^23^ or high fat diet fed obese mice^21^, and reducing body weight gain^21,24^ of obese mice. In humans, we have shown that after a single dose of cinnamaldehyde ingestion, post-prandial energy expenditure and fat oxidation were maintained higher than in placebo^25^. It could be speculated that these effects result from activation of TRPA1^26^. Indeed it has been shown that TRPA1 contribute to thermogenesis^26,27^. Recently, it has been reported that cuminaldehyde, a cumin compound structurally close to cinnamaldehyde, has an anti-obesity effect in diet induced obese rats^28^ and an anti-hyperglycemic effect in a diabetic rat model^29, 30^. Additionally, cumin seed oil has been shown to improve insulin sensitivity of patients with diabetes type II^31^. We speculated that this effect might be due to TRPA1 activation as hypothesized for cinnamaldehyde and investigated the response of TRPA1 to cuminaldehyde as well as to other natural aldehydes, anisaldehyde and tiglic aldehyde.

## Results

### Activation of TRPA1 dependent current by selected compounds

**Figure 1:**
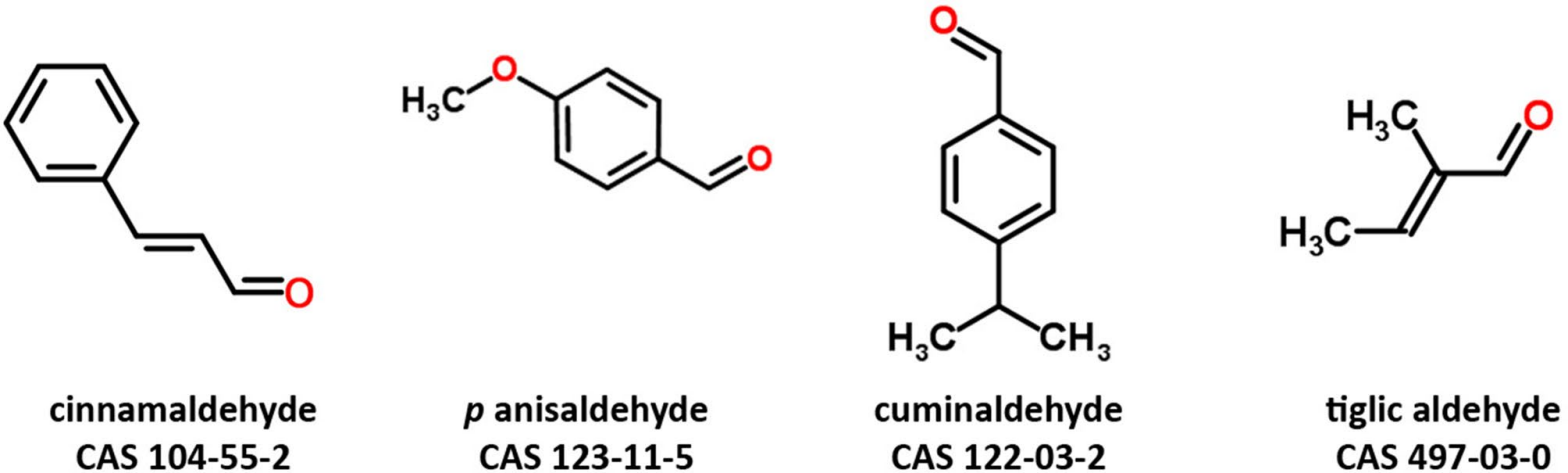
Chemical structure of the different natural compounds tested in vitro.

**Figure 2:**
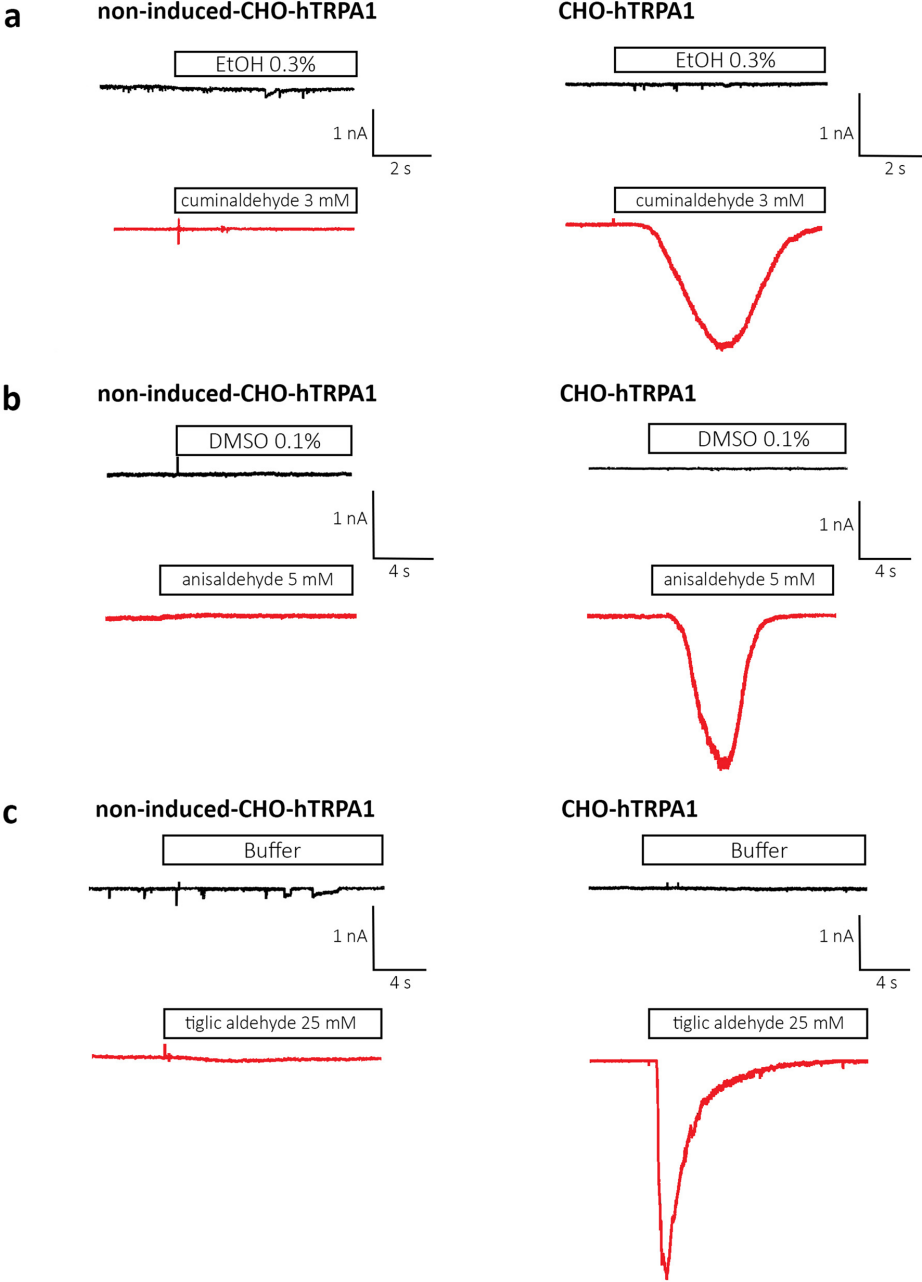
Cuminaldehyde, anisaldehyde and tiglic aldehyde activate a TRPA1-dependent inward current in CHO cells. An inward current is recorded in hTRPA1 expressing cells clamped at resting membrane potential (− 80 mV) under the addition of 5/50 mL of 3 mM cuminaldehyde (**a**), 5 mM anisaldehyde (**b**) or 25 mM tiglic aldehyde (**c**). None of the vehicle buffer (ethanol, DMSO or electrophysiology buffer) at corresponding concentration induced an inward current. None of them elicited any inward currents in the absence of hTRPA1 expression, in non-induced cells.

**Figure 3:**
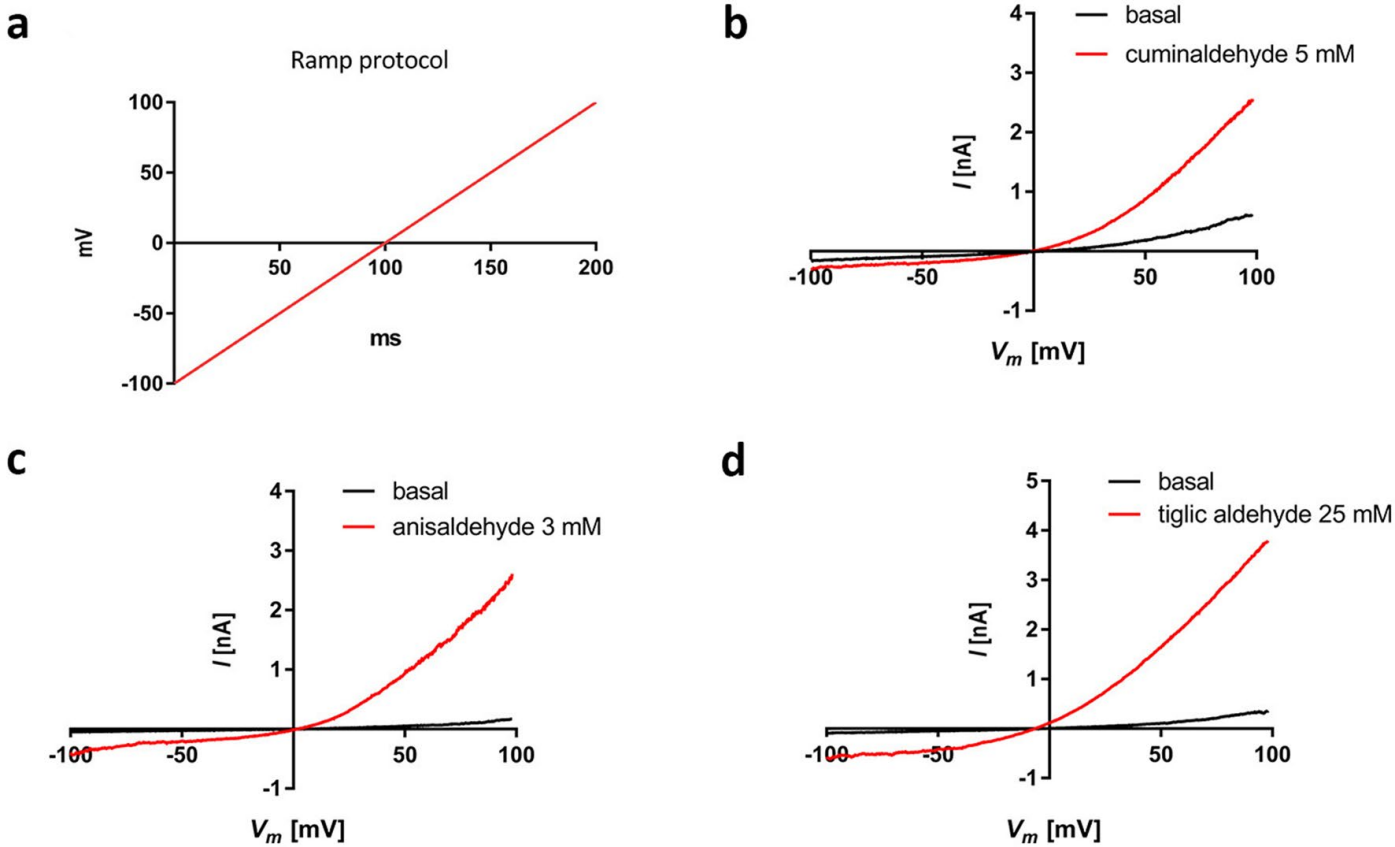
TRPA1-dependent current elicited by cuminaldehyde, anisaldehyde and tiglic aldehyde is voltage-dependent. To set up current–voltage (I–V) relationships of the activation of hTPRA1 by the three compounds, voltage ramp protocols (**a**) were applied. I–V curve under addition of 5/50 mL of cuminaldehyde 5 mM (**b**), anisaldehyde 3 mM (**c**) or tiglic aldehyde 25 mM (**d**) showed the TRPA1 characteristic outward rectifying current and a reverse potential close to zero.

Using whole cell patch-clamp technique in CHO-cells expressing hTRPA1 under the induction of transcription by tetracycline, we tested if the three studied flavors, anisaldehyde, cuminaldehyde and tiglic aldehyde (Fig. 1) activate TRPA1. An inward current was recorded in hTRPA1 expressing cells clamped at resting membrane potential (− 80 mV) under the addition of 5/50 μL of 3 mM cuminaldehyde (Fig. 2a, right panel red trace), 5 mM anisaldehyde (Fig. 2 b, right panel red trace) or 25 mM tiglic aldehyde (Fig. 2c, right panel red trace). The observed inward current was quickly inactivated. None of the vehicle buffer (ethanol, DMSO or electrophysiology buffer) at corresponding concentration induced an inward current in hTRPA1 expressing cells (Fig. 2a–c, right panel black traces). To verify the specificity of these activated currents, non-induced hTRPA1 stably transfected cells where stimulated with same compounds. None of them elicited any inward currents in the absence of hTRPA1 expression (Fig. 2a–c, left panel red traces). To establish the voltage dependence of the activation of hTPRA1 by the three flavors, voltage ramp protocols (Fig. 3a) were applied to set up current–voltage (I–V) relationships (Fig. 3). I–V curve under cuminaldehyde (Fig. 3b), anisaldehyde (Fig. 3c) or tiglic aldehyde (Fig. 3d) showed the TRPA1 characteristic outward rectifying current and a reverse potential close to zero that could be observed under cinnamaldehyde or cold stimulation^32^.

### Dose-dependent activation of TRPA1

**Figure 4:**
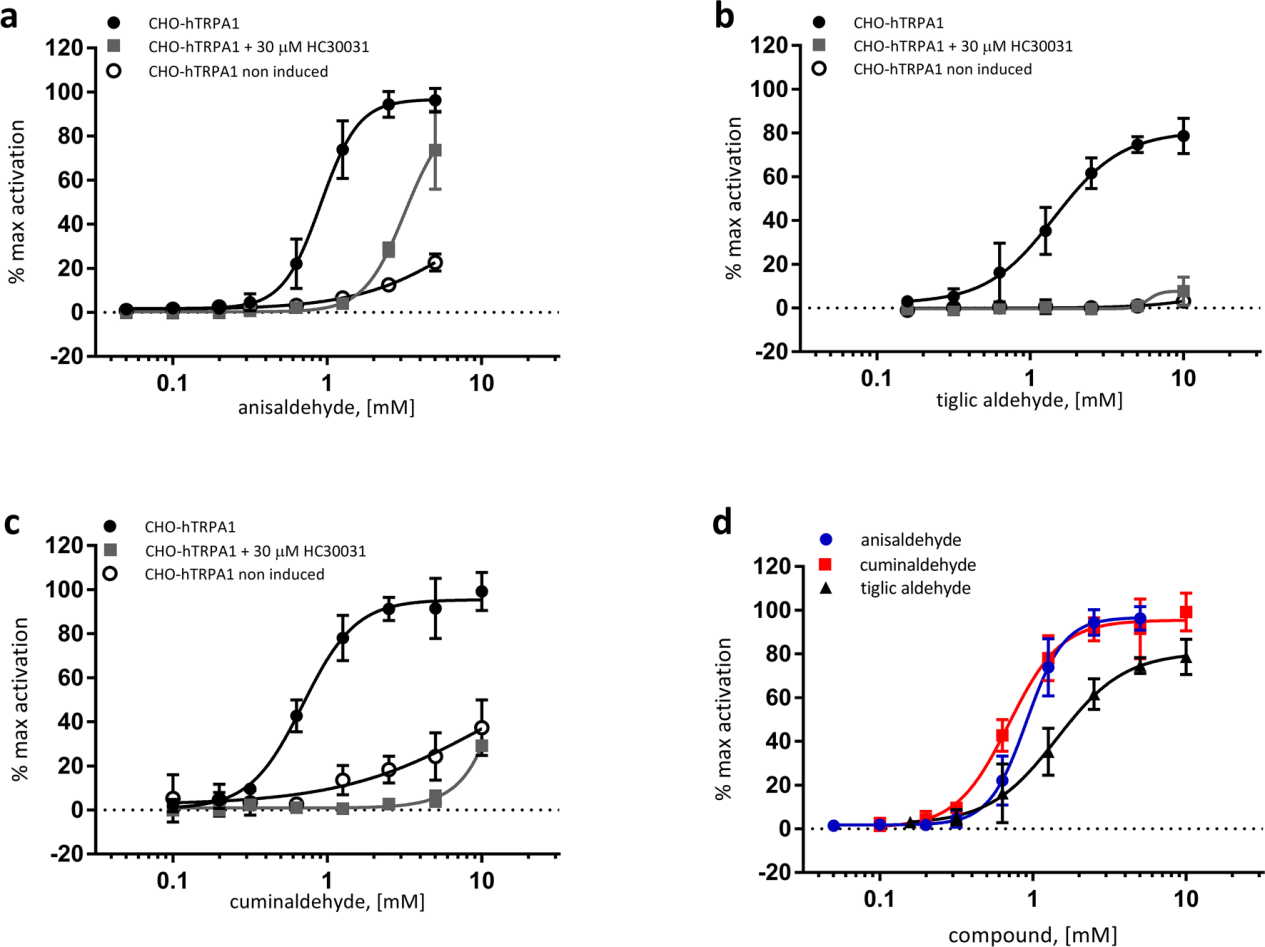
Cuminaldehyde, anisaldehyde and tiglic aldehyde induce TRPA1-dependent calcium release in CHO cells. By ratiometric calcium imaging in hTRPA1 expressing CHO cells, the activation of hTRPA1 by cuminaldehyde, anisaldehyde and tiglic aldehyde (**d**) was measured. Dose–response curves gave half-maximum activation (EC_50_) concentrations of 0.91 mM for anisaldehyde, 0.72 mM for cuminaldehyde and 1.49 mM for tiglic aldehyde. HC030031 (30 μM), a specific TRPA1 antagonist, blocked the responses of anisaldehyde (**a**), tiglic aldehyde (**b**) and cuminaldehyde (**c**). The responses are expressed as “% of max activation”: for CHO-hTRPA1, the response is normalized compared to the response obtained with 100 μM cinnamaldehyde; for Non induced CHO-hTRPA1, the response is normalized compared to the response obtained with 0.3 μM ATP. Error bars are SD and n = 4 wells for each condition.

By ratiometric calcium imaging in hTRPA1 expressing CHO cells, we confirmed the activation of hTRPA1 by cuminaldehyde, anisaldehyde and tiglic aldehyde (Fig. 4d). Doing dose–response curves, we determined half-maximum activation (EC_50_) concentration of 0.91 mM for anisaldehyde, 0.72 mM for cuminaldehyde and 1.49 mM for tiglic aldehyde. Compared to a maximum activation of hTRPA1 obtained by a stimulation with 100 μM of cinnamaldehyde, both anisaldehyde and cuminaldehyde reach equivalent maximum activation with concentrations of 5 mM and 10 mM, respectively. Tiglic aldehyde reach its maximum level of hTRPA1 activation at about 5 mM with represent only 80% of its activation obtained by 100 μM of cinnamaldehyde (Fig. 4d). HC030031 blocked the responses of anisaldehyde (Fig. 4a), tiglic aldehyde (Fig. 4b) and cuminaldehyde (Fig. 4c). Residual activation can be observed at high concentrations of anisaldehyde upon inhibition with HC030031 (Fig. 4a, gray square). This activation could be explained by a non-specific activation of CHO cells since same kind of activation can be observed in non-hTRPA1-expressing CHO (non-induced CHO-hTRPA1) cells (Fig. 4a, open circle). To be noted that at high concentrations, close to 10 mM, non-specific activation of non-hTRPA1-expressing CHO can be observed with anisaldehyde and cuminaldehyde (Fig. 4a,c, open circle). To summarize, by ratiometric calcium imaging, we could show that anisaldehyde, cuminaldehyde and tiglic aldehyde activate hTRPA1 in a dose-dependent manner with lower affinity than reported for cinnamaldehyde, indeed compared to the reported EC_50_ of 61 μM for cinnamaldehyde^32^ we reported here EC_50_ ~ 11, ~ 24 and ~ 24 times higher for cuminaldehyde, anisaldehyde and tiglic aldehyde, respectively. Excepted for tiglic aldehyde, maximum activity comparable to cinnamaldehyde could be reach.

### Specific activation

**Figure 5:**
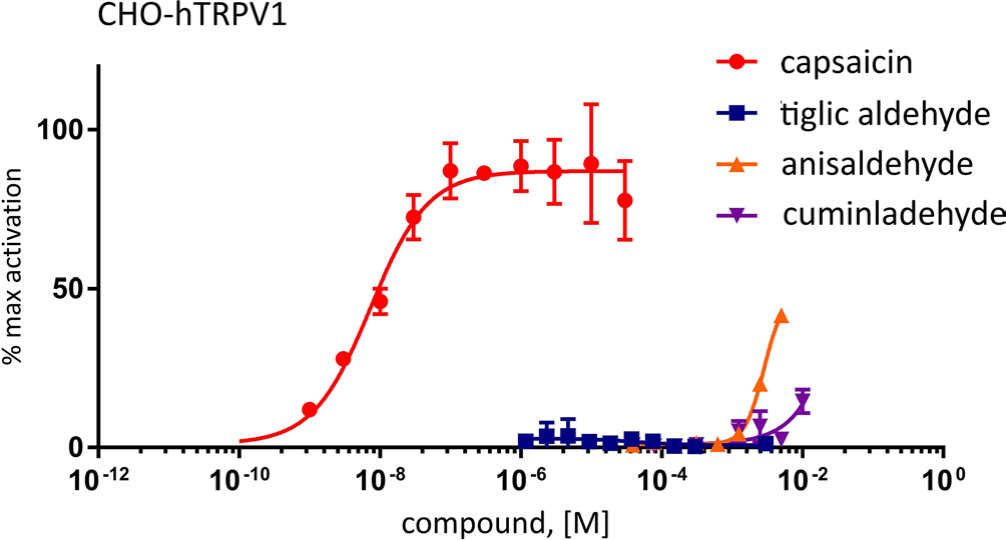
Cuminaldehyde, anisaldehyde and tiglic aldehyde do not activate hTRPV1. By ratiometric calcium imaging on hTRPV1 expressing cells, the activation of hTRPV1 by doses of anisaldehyde, cuminaldehyde and tiglic aldehyde from 1 μM up to 10 mM, was compared to the dose–response of hTRPV1 to capsaicin. Compared to the activation of hTRPV1 by capsaicin, no comparable activation could be observed with any of the compound tested. The responses are expressed as “% of max activation”: in hTRPV1 expressing cells, the response obtained with the compounds is normalized compared to the response obtained with 10 μM capsaicin. Error bars are SD and n = 2 wells for each condition.

Since the selected compounds of this study might produce spicy sensation^33^, we evaluated their capacity to activate the related receptor hTRPV1 activated by pungent ingredients from spices like capsaicin. By ratiometric calcium imaging on hTRPV1 expressing cells, we evaluated the activation of hTRPV1 by doses of anisaldehyde, cuminaldehyde and tiglic aldehyde from 1 μM up to 10 mM, and compared to the dose–response of hTRPV1 to capsaicin (Fig. 5). Compared to the activation of hTRPV1 by capsaicin, no comparable activation could be observed with any of the flavors tested. We could only notice a slight activation upon highest doses of anisaldehyde and cuminaldehyde, which was also observed in hTRPA1 expressing CHO cells and non-induced CHO-hTRPA1 cells (Fig. 4a,c) and interpreted as non-specific to CHO cells. To summarize, no specific activation of hTRPV1 could be recorded under stimulation by anisaldehyde, cuminaldehyde or tiglic aldehyde.

### Activation of DRG neurons in culture

**Figure 6:**
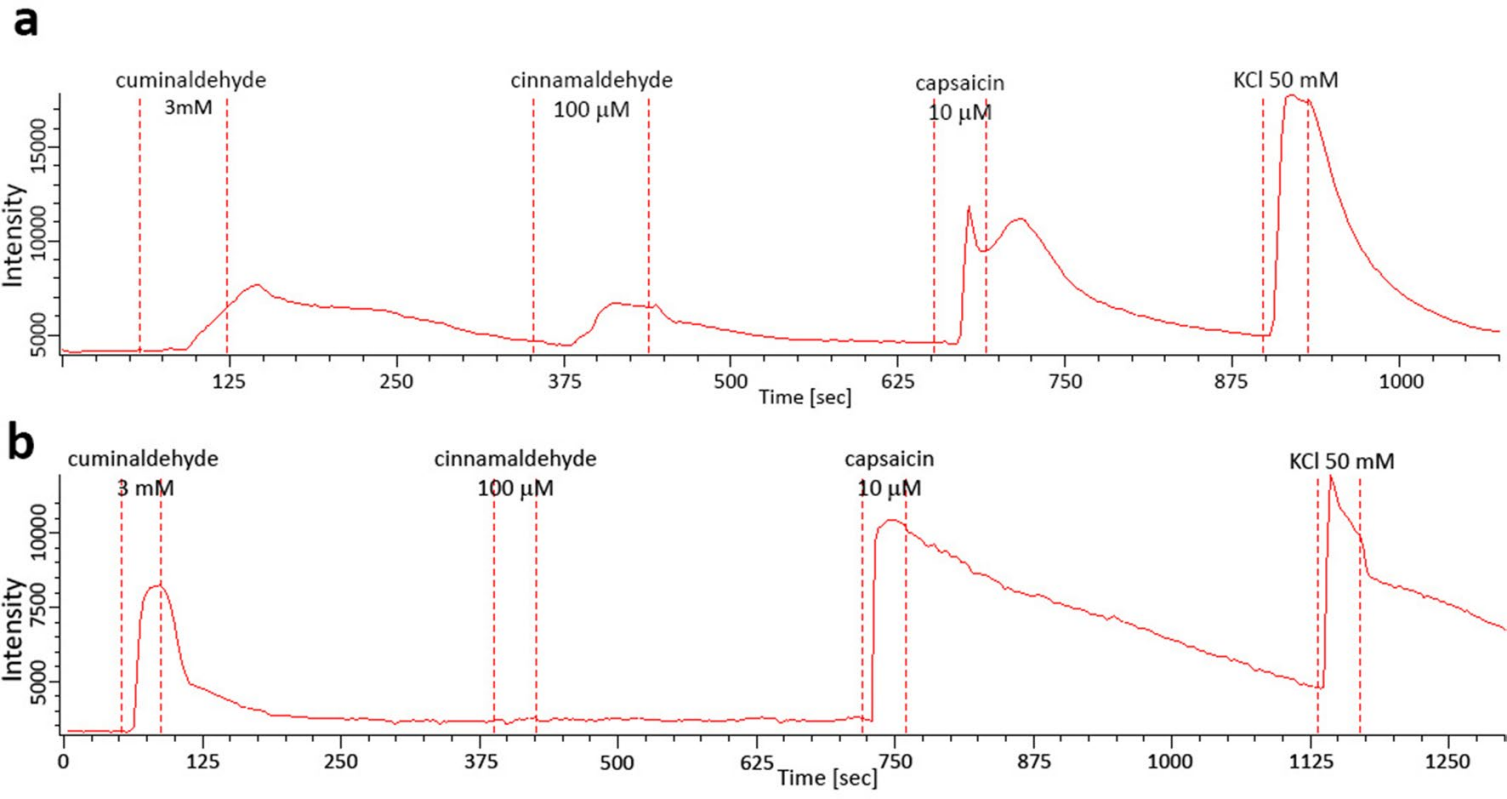
Representative cuminaldehyde responding-cinnamaldehyde responding DRG (**a**) and cuminaldehyde responding-cinnamaldehyde insensitive DRG (**b**). Activation of DRG was measured by calcium imaging single cell assay. Cuminaldehyde activated 17/33 (51%) of recorded DRG neurons, among them 6 were as well activated with cinnamaldehyde (**a**) and 9 not (**b**). Capsaicin 10 μM and KCl 50 mM were applied for neurons characterization and viability check, respectively. Similar profiles of DRG neuron activation were obtained with anisaldehyde and tiglic aldehyde but not shown.

To confirm if the three tested compounds could activate physiologically TRPA1 as cinnamaldehyde^32^, DRG neurons from rats were stimulated with anisaldehyde, cuminaldehyde and tiglic aldehyde and activity recorded by calcium imaging. Buffer control stimulation has been applied as negative control and did not activate DRG neurons (data not shown). To reach DRG neuron responses to anisaldehyde, cuminaldehyde and tiglic aldehyde, we applied 10 mM, 3 mM and 25 mM respectively, where 100 μM of cinnamaldehyde was used to characterize the DRG neurons. Anisaldehyde activated 20/128 (16%) of recorded stimulated neurons, cuminaldehyde 17/33 (51%) (Fig. 6) and tiglic aldehyde 3/11 (27%). Anisaldehyde, cuminaldehyde and tiglic aldehyde activated both cinnamaldehyde sensitive neurons (12/22, 6/12 and 1/2, respectively) and cinnamaldehyde insensitive neurons (8/104, 11/21 and 2/9, respectively). To be noted that all the anisaldehyde responding neurons responded as well to either cinnamaldehyde and/or capsaicin (the TRPV1 agonist); two cuminaldehyde responding neurons and one neuron responding to tiglic aldehyde responded neither to cinnamaldehyde nor to capsaicin.

### Sensory profile of selected ingredients

The selected compounds of this study (Fig. 1) are both reported as flavors and selected for their potential activation of TRPA1. As a confirmation of their capability to stimulate the sensory receptor TRPA1, they have been evaluated in sensory tasting to describe their sensory profile (Table 1). Evaluated concentrations were selected based on safety consideration in the range of flavoring usage in gelatin/pudding food categories. Anisaldehyde evaluated at 47.94 ppm (352 μM) was not reported as tingling but characterized by an herbaceous and anise flavor with a strong persistency of the aromas. Cuminaldehyde at 29 ppm (195 μM) was described as strong in cumin or spicy aroma. Chemesthetic description as irritant, pungent, warm or metallic was associated to the tasted concentration of cuminaldehyde as well as a tingling, burning, metallic sensation in persistency. Tiglic aldehyde at 5 ppm (60 μM) was associated to a tingling or cooling sensation of low overall intensity, an onion after taste and a rubber, fat, almond or syrup-like aroma. Taken together the sensory description show that both cuminaldehyde and tiglic aldehyde are associated to the characteristic chemesthetic sensation produce by the activation of TRPA1 in the trigeminal nerve, which is not the case for anisaldehyde at the given concentration.

**Table 1:** Summary of sensory and in vitro EC_50_ on hTRPA1.

	Reported use level in gelatins/puddings		Sensory description		In vitro EC_50_ on hTRPA1
	ppm	μM	Flavor	Chemesthetic	μM
Anisaldehyde	47.94	352	Herbaceous, anise	Not reported	910
Cuminaldehyde	29.33	195	Cumin, spicy	Irritant, pungent, burning, metallic, tingling,	720
Tiglic aldehyde	5	60	Onion, , fat, almond, syrup-like	Tingling, cooling	1,490

## Discussion

In the present study, we reported that three natural compounds, cuminaldehyde, p-anisaldehyde and tiglic aldehyde from spice’s origin (cumin, anise and onion/garlic, respectively) are able to activate hTRPA1 specifically but with lower affinity than the well described compound of cinnamon oil, cinnamaldehyde. Indeed, we found for cuminaldehyde, p-anisaldehyde and tiglic aldehyde EC_50_ of 0.72 mM, 0.91 mM and 1.49 mM respectively, compared to an EC_50_ of close to 60 μM^32^ for cinnamaldehyde. P-anisaldehyde, as well found in Korean Mint^34^, was also described as a TRPA1 agonist^34^. We report here, for p-anisaldehyde, an EC_50_ of 0.91 mM similar to the published one (0.55 mM)^34^ but, contrary to Moon et al.^34^, we found that p-anisaldehyde reach the similar maximum activation (efficacy) as cinnamaldehyde and can be consider as a full agonist. One risk for agonists of lower affinity is the lack of specificity due to higher concentration needed to get similar lever of receptor activation. By calcium imaging, we observed non-specific activation of TRPA1 at high concentrations of cuminaldehyde and anisaldehyde that might be due to cellular toxicity at high doses and a cellular damage induced calcium release. As many TRPA1 agonists have a non-specific effect reported on TRPV1^17^, we verified the cross-activation on TRPV1 (Fig. 5) and confirmed the specificity of these agonists on TRPA1 vs. TRPV1. However, we cannot exclude other non-evaluated non-specific effects at high concentration.

Cuminaldehyde, p-anisaldehyde and tiglic aldehyde activated subpopulations of sensory neurons from rat DRG. We observed that the concentrations used to obtain recordable responses in DRG neurons were higher than in hTRPA1 expressing CHO cells. This might be explained by species differences in the pharmacology of primate and rodent TRPA1 already described for several compounds^35^. It is worth noting also that diverse populations of neurons responded to anisaldehyde, cuminaldehyde or tiglic aldehyde: neurons responding to both or only capsaicin or cinnamaldehyde, or neurons responding to neither capsaicin or cinnamaldehyde. The diverse profile of DRG neurons responding to these three tested compounds might reflect the already described high neural diversity in DRG^36^ or trigeminal ganglion neurons^37^ and variance in gene expression profile among it. Still, a functional role for each neural profile is not clear.

Sensory features of these compounds are not predictive of their pharmacological activities on TRPA1. Indeed, even though cuminaldehyde and tiglic aldehyde were tasted at concentrations below EC_50_, their sensory properties were associated to chemesthetic descriptive such as tingling, burning or metallic that could be attributed to the stimulation of hTRPA1 in trigeminal fibers, which was not the case for anisaldehyde. The differences in chemesthetic sensation might be due to the degradation in the saliva or their capability to diffuse into the epithelium. Discrepancy in sensory properties and in vitro activity on a TRP channel has been previously described for TRPA1^38^. Indeed, the in vitro recording of only TRPA1 activity in a heterologous cellular expression was not able to fully explain the perception of sensory irritation produced by an environmental pollutant. The sensory perception should rather result from the activation of a class of receptors and/or cross-interactions between them^38^, which might be the case as well for the coding of chemesthetic sensation produced by chemicals from spices.

As cinnamaldehyde^21^, cuminaldehyde^28^ after 5 or 6 weeks, respectively, of daily ingestion, has shown to promote reduction of body weight gain and lower level of glucose in blood of diet induced obese rodents. Additionally to cuminaldehyde, Haque et al.^28^ evaluated the impact of thymol on the metabolism of high fat diet induced obese rats which show to have as well potent anti-obesity effect while not affecting glycaemia. They observed effects that were more significant when cuminaldehyde and thymol were combined than thymol or cuminaldehyde alone. As both cinnamaldehyde^32^ and thymol^39^ are known to activate TRPA1 and this study reports as well the activation of TPRA1 by cuminaldehyde, it can be speculated that their metabolic effect is partially due to TRPA1 activation, even though nor the effect of cinnamaldehyde neither of cuminaldehyde and thymol on metabolism are understood. Additionally, concurring data can be found in the characterization of other plant extracts activity, as gingerol shown both anti-obesity action^40^, and TRPA1 activation^41^. However, the proposed anti-obesity or anti-hyperglycemic mechanisms of action of gingerol and cuminaldehyde are through the inhibition of α-amylase^40^ or α-glucosidase, reducing the intestinal absorption of carbohydrates, and aldose reductase^42^ inhibition, respectively. Reinforcing the hypothesis that alternative explanation than activation of TRPA1 may occur in the metabolic effects of cinnamaldehyde and cuminaldehyde is the comparison of in vivo efficient doses to their receptor’s potency. Indeed, doses with in vivo impact on metabolism in rodent were, for cuminaldehyde reported by Haque et al.^28^ and for cinnamaldehyde reported in our previous study^21^, respectively 12 mg/kg bw/day and 250 mg/kg bw/day; as the potency of cinnamaldehyde on TRPA1 is higher than cuminaldehyde, we would expect, if the metabolic effects would be due to TPRA1, to have same in vivo effect with lower doses of cinnamaldehyde compared to cuminaldehyde.

Cuminaldehyde demonstrated as well a glucose dependent insulin secretagogue activity in diabetics rats^30^. Interestingly the antidiabetic drug glibenclamide induces insulin release via inhibition of K(ATP) channels in pancreatic β-cell, but also activates TRPA1^43^. As the activation of TRPA1, expressed in pancreatic β-cells, induces insulin release^13^, it might be speculated that the in vivo secretagogue effect of cuminaldehyde is due to TRPA1 stimulation.

It has been as well shown in human, that after a single dose ingestion of cinnamaldehyde energy expenditure and fat oxidation are maintained higher than in placebo condition^25^. Moreover, chronic ingestion of cinnamaldehyde reduces visceral adipose tissue in rodents and increase UCP1 expression in brown adipose tissue (BAT)^24^. It has been proposed that TRPA1 could increase metabolism through cold sensing and thermogenesis stimulation in BAT^44^. It might be as well speculated that long term anti-diabetic effects of TRPA1 agonists as cinnamaldehyde and cuminaldehyde are due to increased energy expenditure and fat oxidation.

As cinnamaldehyde or cuminaldehyde, several natural solutions like capsaicin from red chili or curcumin^45^ have been proposed for the improvement of obese or diabetic conditions. Natural solutions with history of food usage could easily be included in the daily diet and complement the efficacy of drug therapy or help, associated to lifestyle modifications, to prevent obesity and diabetes. More broadly, natural solutions could be foreseen for all TRPA1 associated improvement of health disorder that could be associated, as vasodilatation^46^ or improvement of swallowing disorders in elderly^10^. However, regarding some discrepancy between potency of TRPA1 agonist and their in vivo effective doses on metabolism, more pre-clinical evidence are needed to confirm the potential of this pathway to target metabolic activities.

Additionally, clinical evidences and assessment of side effects, as noxious irritation^47^ related to TRPA1 stimulation, are needed to conclude on the real opportunity to use TRPA1 agonists as complement to preventive or therapeutic approaches.

## Material and methods

### Material

#### Chemicals

 p-Anisaldehyde #W267007, Cuminaldehyde #W234109, Tiglic aldehyde #W340707 and HC030031 #H4415 were purchased at Sigma Aldrich.

#### TRPA1 cell line

 CHO Tetracycline-inducible cells, stably transfected with human TRPA1 channel, were purchased from ChanTest Corp. (Cleveland, USA). Cells were grown in a humidified 5% CO2 incubator at 37 °C, using a Ham’s F12 medium (Life Technologies) supplemented with 10% Fetal Bovine Serum (AMIMED, BioConcept), 100units/ml Penicillin–Streptomycin (Sigma-Aldrich), 0.01 mg/ml Blasticidin S HCl (Life Technologies), and 0.4 mg/ml Zeocin (Life Technologies) in 75 cm^2^ tissue culture flasks (Corning Life Sciences). Cells were split every 3–4 days, when 80% confluence was reached.

#### TRPV1 cell line

 CHO cells, stably transfected with human TRPV1 channel were purchased from B’SYS GmbH. Cells were grown in a humidified 5% CO_2_ incubator at 37 °C, using a Ham’s F12 medium (Life Technologies) supplemented with 10% Fetal Bovine Serum (AMIMED, BioConcept), 100 units/ml Penicillin–Streptomycin (Sigma-Aldrich) in 75 cm^2^ tissue culture flasks (Corning Life Sciences). Antibiotic of selections was also added in this medium: Geneticin 500 μγ/ml (Gibco). Cells were split every 3–4 days, when 80% confluence was reached.

#### Rat Dorsal Root Ganglion neuron (DRG)

DRG neurons were cultured as describe by the supplier (Lonza cat.number R-DRG-505). Briefly, DRG were thawed in fully supplemented PNGM media and plated on glass coverslips previously coated with poly-D-lysine and Laminin. After 4 h at 37 °C, mitotic inhibitors were added.

### Calcium imaging

#### 96-well assay

 The day before the experiment cells were seeded to reach the density of 25′000 cells per well in sterile 96-well assay plates (Corning Life Sciences) using a Ham’s F12 medium supplemented with 10% Fetal Bovine Serum, 100 units/ml Penicillin–Streptomycin and 1 μγ/ml Tetracycline Hydrochloride (Sigma Aldrich) to allow the expression of the human TRPA1. For the control (CHO background cells without TRP channels) and the CHO_hTRPV1, cells were seeded in the same conditions but without Tetracycline. Plates were let 18–24 h in a humidified 5% CO2 incubator at 37 °C. The day of the experiment cells were loaded with a 2 mM Fura-2AM (Life Technologies) and 0.04% Pluronic acid F-127 (Life Technologies) solution and incubated 45 min in a humidified 5% CO2 incubator at 37 °C. Then the cells were washed with calcium buffer (137 mM NaCl, 4 mM KCl, 1.8 mM CaCl_2_, 1 mM MgSO_4_, 10 mM Hepes, 10 mM (D) Glucose, buffered at pH 7.4) and let 15 min in a humidified 5% CO2 incubator at 37 °C. The 96-well plates were then placed into the plate reader (FLEXstation; Molecular Devices) which added the compounds previously diluted in the calcium buffer from a source plate to the cell plate and measured changes in Ca^2+-^induced fluorescence intensity (FI) of dye at F340/380 (_ex1_340 nm;_ex2_380nm; _em _ 510 nm).

The fluorescence intensities (FI) were normalized as described below:

$$\% {\text{ activation}} = {{\left( {{\text{FIX}} - {\text{FI buffer}}} \right)} \mathord{\left/ {\vphantom {{\left( {{\text{FIX}} - {\text{FI buffer}}} \right)} {\left( {{\text{FI }}\max - {\text{FI buffer}}} \right)}}} \right. \kern-\nulldelimiterspace} {\left( {{\text{FI }}\max - {\text{FI buffer}}} \right)}}$$

FI_max_ being the fluorescence of agonists used as positive controls at a concentration giving maximum activation (data not shown):

$$\begin{aligned} & {\text{Cinnamaldehyde }}\left( {\text{Sigma Aldrich}} \right) \, 100\;{\text{mM for CHO\_hTRPA}}1 \\ & {\text{Capsaicin }}\left( {\text{Sigma Aldrich}} \right) \, 1\;{\text{mM for CHO\_hTRPV}}1 \\ & {\text{ATP }}\left( {\text{Sigma Aldrich}} \right) \, 0.3\;{\text{mM for control }}\left( {{\text{CHO}}} \right) \\ \end{aligned}$$

#### Single Cell assay

 Single cell calcium imaging experiments were performed on an AXIO Observer. D1 (Zeiss), a digital camera ORCA-Flash4.0 (Hamamatsu), a High Speed Polychromator System VisiChrome and acquired by VisiView software (Visitron Systems GmbH). The day of experiment, DRG were loaded during 45 min in Fluo 4 containing probenecid (Fluo-4 NW Calcium Assay Kit, Molecular Probes, cat.number F36206). Coverslips were mounted in a bath-imaging chamber RC-25F, with VC-8 Valves Controller through a dual automatic temperature controller TC-344B (Warner Instruments). DRG are continuous perfused at 37 °C with HBSS, 20 mM HEPES, 2 mM CaCl2, pH7.4 (Sigma). DRG were first perfused 10 min for washing, and stimulations were performed during 50 s with 25 mM Tiglic Aldehyde, 5 mM p-Anisaldehyde, 3 mM Cuminaldehyde, 100 µM Cinnamaldehyde (Sigma) and 10 µM Capsaicin (Sigma). 50 mM KCl (Sigma) was used as the positive control. Fluorescence was measured using excitation at 494 nm and emission at 516 nm.

### Electrophysiology

The day before the experiment TRPA1 cells were induced using a Ham’s F12 medium supplemented with 10% Fetal Bovine Serum, 100 units/ml Penicillin–Streptomycin and 1 μγ/ml Tetracycline Hydrochloride (Sigma Aldrich) to allow the expression of the human TRPA1. The day of the experiment cells were harvested using 5 min treatment with Accutase (Sigma Aldrich) and kept in suspension in extracellular medium (140 mM NaCl, 4 mM KCl, 1 mM MgCl2, 2 mM CaCl2, 5 mM D-glucose monohydrate and 10 mM Hepes/NaOH, ph 7.4, 298 mOsmol).

All electrophysiological data were collected in the whole-cell configuration using the Port-a-Patch automated system (Nanion). Intracellular solution is 50 mM CsCl, 10 mM NaCl, 60 mM Cs-Fluoride, 20 mM EGTA and 10 mM Hepes/CsOH, ph 7.2, 295 mOsmol and extracellular solution is 140 mM NaCl, 4 mM KCl, 1 mM MgCl_2_, 2 mM CaCl2, 5 mM d-glucose monohydrate and 10 mM Hepes/NaOH, ph 7.4, 298 mOsmol. Microchips (Nanion) of 2–5 mOhm were used. Voltage-clamp recordings were obtained using EPC 10 patch-clamp amplifier (HEKA) and PatchMaster software (HEKA) and PatchControl software (Nanion). Recordings were performed at room temperature at an holding potential of − 80 mV. Series resistances, and fast and slow capacitance transients were compensated by the patch-clamp amplifier. Only cells with leak currents below 100 pA and resistance around 1 GOhm were analyzed.

### Sensory evaluation

For safety concern, the doses of compounds used for technical sensory evaluation were the maximum reported use level in food categories related to gelatins and pudding by Burdock^33^. Anisaldehyde was evaluated at 47.94 ppm (352 μM), Cuminaldehyde at 29 ppm (195 μM) and Tiglic aldehyde at 5 ppm (60 μM) in TUC at nectar viscosity (Resource ThickenUp Clear 2.4 g in 200 ml of water). Compounds were first prepared as stock solution in ethanol (Tiglic aldehyde: 20 mg/ml, Cuminaldehyde: 116 mg/ml, p-anisaldehyde: 192 mg/ml). Seven voluntary panelists were asked to assess the sensory characteristics of the samples and conclude on the most obvious shared sensory descriptors.
